# Altered Ca^2+^ responses and antioxidant properties in Friedreich's ataxia-like cerebellar astrocytes

**DOI:** 10.1242/jcs.263446

**Published:** 2025-01-09

**Authors:** Chiara Marullo, Laura Croci, Iris Giupponi, Claudia Rivoletti, Sofia Zuffetti, Barbara Bettegazzi, Ottavio Cremona, Paola Giunti, Alessandro Ambrosi, Filippo Casoni, Gian Giacomo Consalez, Franca Codazzi

**Affiliations:** ^1^ Division of Neuroscience, IRCCS San Raffaele Scientific Institute, 20132 Milan, Italy; ^2^ Faculty of Medicine and Surgery, Università Vita-Salute San Raffaele, Milan, Italy; ^3^ Ataxia Centre, Department of Clinical and Movement Neurosciences, UCL Queen Square Institute of Neurology, London WC1N 3BG, UK

**Keywords:** Friedreich's ataxia, Cerebellar astrocytes, Oxidative stress, Ca^2+^ signalling, Mitochondria

## Abstract

Friedreich's ataxia (FRDA) is a neurodegenerative disorder characterized by severe neurological signs, affecting the peripheral and central nervous system, caused by reduced frataxin protein (FXN) levels. Although several studies have highlighted cellular dysfunctions in neurons, there is limited information on the effects of FXN depletion in astrocytes and on the potential non-cell autonomous mechanisms affecting neurons in FRDA. In this study, we generated a model of FRDA cerebellar astrocytes to unveil phenotypic alterations that might contribute to cerebellar atrophy. We treated primary cerebellar astrocytes with an RNA interference-based approach, to achieve a reduction of FXN comparable to that observed in individuals with FRDA. These FRDA-like astrocytes display some typical features of the disease, such as an increase of oxidative stress and a depletion of glutathione content. Moreover, FRDA-like astrocytes exhibit decreased Ca^2+^ responses to purinergic stimuli. Our findings shed light on cellular changes caused by FXN downregulation in cerebellar astrocytes, likely impairing their complex interaction with neurons. The potentially impaired ability to provide neuronal cells with glutathione or to release neuromodulators in a Ca^2+^-dependent manner could affect neuronal function, contributing to neurodegeneration.

## Teaser

**Summary:** FRDA-like astrocytes display some typical features of the disease, such as oxidative stress and depletion of glutathione. Moreover, FRDA-like astrocytes exhibit decreased Ca^2+^ responses to purinergic stimuli, likely impairing their complex interaction with neurons.

## INTRODUCTION

Friedreich's ataxia (FRDA), a severe progressive neurodegenerative disorder, is the most common inherited autosomal recessive ataxia, affecting approximately one in every 50,000 individuals. In most cases, FRDA is caused by biallelic expansion of a naturally occurring GAA repeat in the first intron of the *FXN* gene, resulting in a decrease in its transcription (Campuzano et al., 1996; Keita et al., 2022; Vicente-Acosta et al., 2022). The onset of the disease is observed between the first and the second decade of life and it is inversely correlated with the number of GAA repeats in the *FXN*. Symptoms are progressive and result in severe disability within 10–15 years. Premature death most often occurs because of severe cardiomyopathy (Koeppen, 2011).

FRDA is primarily caused by the deficiency of frataxin (FXN), a ubiquitously expressed mitochondrial protein involved in the synthesis of iron–sulfur clusters, by facilitating their introduction to enzymes containing this prosthetic group (among others, enzymes involved in oxidative phosphorylation and the Krebs cycle). Consequently, FXN deficient cells display a dysregulation of cellular iron metabolism, an increase of reactive oxygen species (ROS) production and, more broadly, of oxidative stress, besides a decreased ATP production and cellular dysfunction (Martelli and Puccio, 2014; Pastore and Puccio, 2013).

Although FXN depletion is ubiquitous in FRDA tissues, the cells most clinically affected include large sensory neurons of the dorsal root ganglia (DRG), which manifests as degeneration of ascending dorsal columns, as well as dentate nucleus cells of the cerebellum and other neurons within the retina and the brain. Cardiomyocytes and pancreatic islet cells are also affected. These features contribute to a multisystemic disorder characterized by neurological symptoms, such as slowly progressive ataxia of gait and limbs, spasticity, loss of proprioceptive sensation and deep tendon reflexes, dysarthria, visual dysfunction and hearing loss. Additionally, individuals with FRDA might experience progressive hypertrophic cardiomyopathy, musculoskeletal features and an elevated risk of diabetes (Harding et al., 2020; Lynch et al., 2021; Pandolfo, 2009).

Although the neurodegenerative features of FRDA primarily stem from pathophysiological changes in peripheral and central nervous system neurons, FXN depletion might also impact non-neuronal cells (Koeppen, 2011), potentially contributing to disease pathogenesis. Among these cells, astrocytes stand out as the most abundant glial cell type in the nervous system (Sofroniew and Vinters, 2010) and have been implicated in various neurodegenerative disorders, including Huntington's disease (HD), amyotrophic lateral sclerosis (ALS), multiple sclerosis (MS), Alzheimer's disease (AD) and Parkinson's disease (PD) (Sofroniew and Vinters, 2010; Stoklund Dittlau and Freude, 2024; Yuan et al., 2023). Astrocytes are widely recognized for their key contributions to neuronal homeostasis. They provide essential nutrients and metabolic support to neighbouring neurons, while also playing a key role in buffering metabolic waste, K^+^ and H^+^ ions along their complex processes. Moreover, astrocytes are involved in the handling of neurotransmitters and neuromodulators, as they can uptake and release these signalling molecules, modulating synaptic activity over short and long distances (Navarrete and Araque, 2014). In particular, the ability of astrocytes to remove the excess of glutamate from synaptic clefts plays a crucial role in preventing excitotoxicity, a process implicated in several neurological disorders (Belanger and Magistretti, 2009). Moreover, during inflammatory and oxidative conditions, typical of FRDA, astrocytes undergo an activation process that can have either detrimental or neuroprotective effects. On one hand, activated astrocytes might alter their neuronal support function and release toxic factors, contributing to neuronal death. On the other hand, it is possible that activated astrocytes increase their antioxidant defences and enhance their ability to handle iron, thus exerting a neuroprotective role (Sofroniew and Vinters, 2010; Macco et al., 2013; Pelizzoni et al., 2013). Of note, GFAP, a marker of astrocyte activation, has also been detected in the plasma of individuals with FRDA (Zeitlberger et al., 2018).

Interestingly, selective deletion of the *FXN* gene in glial cells of *Drosophila melanogaster* causes FRDA-like symptoms comparable to those of the whole-body knockout flies (Navarro et al., 2010). Furthermore, deletion of *Fxn* during development in mouse cells with an active GFAP promoter, predominantly astrocytes, induces severe ataxia and premature death, primarily impacting the survival of cerebellar astrocytes (Franco et al., 2017). Recent *in vitro* studies also showed that FXN knockdown in human astrocytes caused several alterations in these cells (e.g. decreased viability and proliferation, and mitochondrial dysfunction) and directed the activation process towards a pro-inflammatory and neurotoxic phenotype, contributing to neuron degeneration (Loria and Diaz-Nido, 2015; Vicente-Acosta et al., 2022).

However, in the encephalon, the major neuropathological changes linked to FRDA are observed in the cerebellum, a crucial brain region for motor control and cognitive function. FRDA cerebellar signs manifest as progressive atrophy of the dentate nucleus, primarily due to degeneration of large glutamatergic neurons (Koeppen et al., 2011; Selvadurai et al., 2018). Despite this, only limited information is available on cerebellar astrocytes, the consequences of FXN depletion on their phenotype, and their possible contribution to cerebellar dysfunction.

In the present study, we established a model of FRDA cerebellar astrocytes (FRDA-like astrocytes) through *FXN* downregulation, using an RNA interference-based approach in primary cultures of mouse cerebellar astrocytes. Our findings reveal significant functional alterations in FRDA astrocytes, including higher mitochondrial ROS production and increased consumption of the antioxidant molecule glutathione, as well as dysregulation of Ca^2+^ homeostasis. These results shed light on FRDA pathophysiology in the cerebellum and might contribute to the development of new strategies for disease treatment.

## RESULTS

### Downregulation of frataxin expression in cerebellar astrocytes transduced with selective shRNAs

**Fig. 1.**
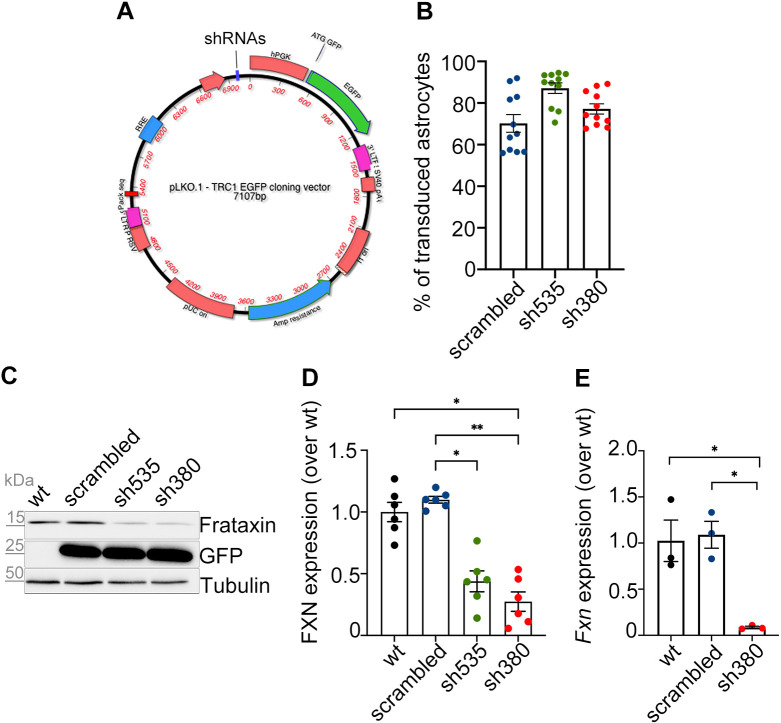
**Frataxin knockdown in cerebellar astrocytes.** (A) Representation of the pLKO.1-TRC cloning vector encoding one of three different shRNAs (either scrambled, sh535 or sh380) and the enhanced green fluorescent protein (EGFP). The expression of shRNAs is driven by the hU6 promoter. Ampicillin resistance was used for plasmid selection. (B) The graph shows the percentage of lentivirally transduced astrocytes assessed by high-throughput microscopy; *n*=10,107 cells for scrambled, 9771 for sh535, and 8263 for sh380, from five biological replicates; each dot represents the mean percentage of transduced cells in a single culture well. (C) Western blot of protein lysates derived from wild-type (wt), scrambled-, sh535- and sh380-transduced astrocytes, immunostained with antibodies detecting FXN (15 kDa). GFP (25 kDa) was used to assess the transduction efficiency; α-tubulin (50 kDa) was used as a loading control. (D) FXN protein levels, normalized to α-tubulin levels, in scrambled-, sh535-, and sh380-transduced astrocytes, compared to wt astrocytes. Data are expressed as mean±s.e.m.; *n*=6; *P<0.05, **P<0.01 (Kruskal–Wallis test). (E) *Fxn* mRNA levels, normalized to β-actin, in scrambled- and sh380-transduced astrocytes, determined by RT-qPCR compared to wt. Data are expressed as mean±s.e.m.; *n*=3 *P<0.05 (Brown–Forsythe and Welch one-way ANOVA tests with multiple comparisons).

To characterize the impact of FXN deficiency on cerebellar astrocytes, we initially established primary astrocytic cultures and defined the conditions for transduction, using lentiviral particles encoding two validated shRNAs, TRCN0000178380 and TRCN0000198535 (hereafter referred to as sh380 and sh535, respectively). The pLKO.1 SHC002 vector, hereon referred to as scrambled, was chosen as the control for the nonspecific effect of lentiviral transduction. To visualize the transduced cells, we replaced one of the two antibiotic resistance sequences, the puromycin cassette, with the GFP coding sequence (see Fig. 1A). The transduction efficiency, as quantified by the ArrayScan instrument, was ∼70% for the scrambled construct, 87% for sh535 and 77% for sh380 (evaluated by GFP positivity on >10,000, >9000 and >8000 cells, respectively; Fig. 1B). We assessed the effect of our knockdown approach on frataxin expression, at both protein and transcript levels. Western blot (WB) analysis, performed 7 days after lentiviral transduction, revealed a reduction of FXN levels to ∼50% with sh535 and 30% with sh380, compared to the levels in untransduced (WT) or control (scrambled-transduced) astrocytes (Fig. 1C,D). The degree of protein downregulation achieved using sh380 matches FXN levels observed in tissues from individuals with FRDA (Gottesfeld et al., 2013). Given the lower efficiency of sh535, all subsequent experiments were conducted using sh380 only, hereafter referred to as sh380-transduced or FRDA-like astrocytes. To compare protein and mRNA downregulation, we performed an RT-qPCR on astrocyte mRNAs, demonstrating a sharp decrease in *Fxn* transcript levels (Fig. 1E). The observed differences in protein versus mRNA downregulation are likely explained by the long half-life (Li et al., 2008) of the FXN protein, which persists even 1 week after lentiviral infection. Again, WT and scrambled-transduced astrocytes displayed comparable *Fxn* transcript levels (Fig. 1E).

Having established an *in vitro* model of FRDA-like cerebellar astrocytes, we evaluated the impact of FXN downregulation on their morphological features. Indeed, previous studies (Pastore et al., 2003) have shown cytoskeletal alterations in fibroblasts from individuals with FRDA, characterized by increased glutathionylation of actin filaments and disorganization of microfilaments. In our study, we performed an immunofluorescence analysis using an anti-GFAP antibody. Our results revealed GFAP positivity of ∼43% in control astrocytes, and 48% in FRDA-like astrocytes (Fig. S1A,B). However, the expression of S100β, a Ca^2+^-binding protein abundantly expressed in astrocytes, was scored in ∼99% of the cells, both scrambled and sh380 transduced (Fig. S1C,D). The heterogeneity observed in our primary cultures reflects the highly diverse morphologies of cerebellar astrocytes observed both in culture and *in vivo* models (Buffo and Rossi, 2013; Cerrato, 2020). However, cellular morphology was not overtly affected by changes in FXN levels and remained comparable in scrambled and FRDA-like astrocytes (Fig. S1A,C).

### Effects of FXN downregulation on the oxidative status of cerebellar astrocytes

**Fig. 2.**
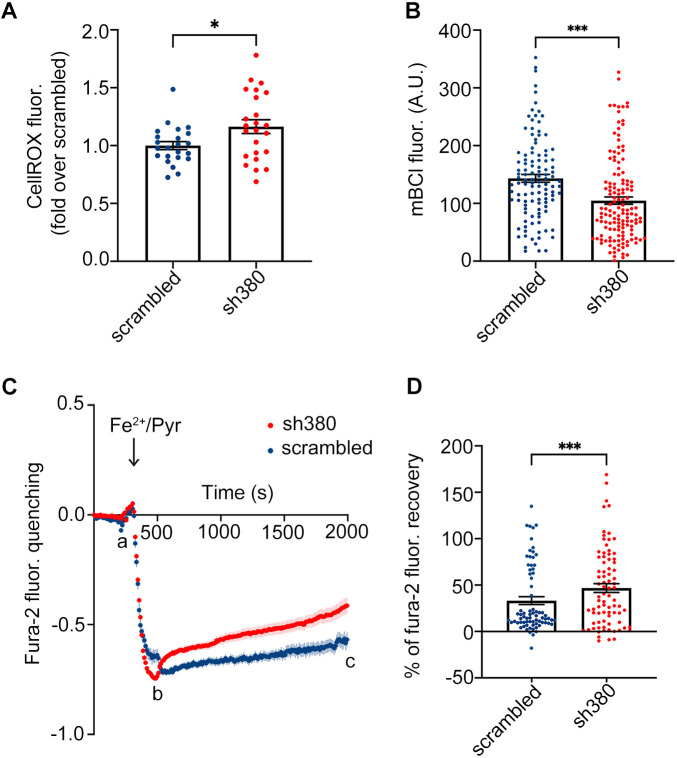
**Increased ROS levels and impaired antioxidant defences in FRDA-like astrocytes.** (A) Quantification of ROS production, analysed at the single-cell level by high-throughput microscopy, and evaluated by CellROX fluorescence in sh380- compared to scrambled-transduced cells. Each dot represents the average of an individual culture well, from three biological replicates. Data are expressed as mean±s.e.m. *P<0.05 (Mann–Whitney test). (B) Measurement of GSH content, estimated in single cells by mBCl fluorescence, in scrambled and sh380-transduced astrocytes. Each dot represents the fluorescence levels recorded at the plateau phase (20 min after mBCl administration) in each individual cell. Data from three biological replicates, are expressed as mean±s.e.m.; *n*=90 cells for scrambled, 179 for sh380; ***P<0.001. (C) Fura-2 fluorescence kinetics, analysed at the Ca^2+^-insensitive excitation wavelength of 356 nm, upon administration of Fe^2+^ (100 μM) in the presence of pyrithione, an iron ionophore (20 μM), in scrambled- and sh380-transduced astrocytes (blue and red, respectively). Fura-2 basal fluorescence (a) was quenched by Fe^2+^ entry induced by Fe^2+^/Pyr administration (b); the oxidation of Fe^2+^ to Fe^3+^ eventually caused fluorescence recovery (c). Data from three biological replicates are expressed as mean±s.e.m.; *n*=74 cells for scrambled, 81 for sh380. (D) Percentage of fura-2 fluorescence recovery in scrambled- and sh380-transduced astrocytes. Data correspond to the ratio of fluorescence recovery to fluorescence quenching [(c−b)/(b−a)]. Each dot represents the fluorescence of a single cell. Data from three biological replicates are expressed as mean±s.e.m.; *n*=74 cells for scrambled, and 81 for sh380; ***P<0.001. Statistical analysis in B and D was performed with a linear mixed effect model with nested random effects on optical microscopy fields within the experiment. A.U., arbitrary units.

A distinctive hallmark of FRDA is represented by oxidative stress, which has been observed in animal and cellular models, as well as in tissues from individuals with FRDA (Lupoli et al., 2018; Pilotto et al., 2024). To assess whether our astrocytic model recapitulates the pathophysiology of FRDA, scrambled- and sh380-transduced astrocytes were loaded with Cell-ROX Orange, a mitochondrial probe whose fluorescence depends on ROS-mediated oxidation. Our results were obtained using high-throughput microscopy to extend the analysis to a large number of cells (Vannocci et al., 2018). Our results showed a significant increase in ROS levels in FRDA-like astrocytes compared to in controls (Fig. 2A).

As the primary antioxidant defence in glial cells is represented by reduced glutathione (GSH), we assessed whether increased ROS accumulation could interfere with GSH levels. To this end, cerebellar astrocytes were loaded with monochlorobimane (mBCl), a probe that fluoresces upon conjugation with GSH (Bettegazzi et al., 2019). The fluorescence was quantified after 20 min, when the reaction reached the plateau phase. In FRDA-like astrocytes, the final fluorescence level was significantly lower compared to that in control astrocytes, indicating GSH consumption, in a likely attempt to counteract the ROS increase caused by FXN deficiency (Fig. 2B).

Based on this evidence, we asked whether the decrease in GSH levels scored in FRDA-like astrocytes could lead to an exhaustion of their antioxidant defences. To this end, cerebellar astrocytes were loaded with fura-2 and exposed to an acute Fe^2+^ overload (100 μM) in the presence of pyrithione. The unique property of fura-2, whose fluorescence is selectively quenched by Fe^2+^ but not Fe^3+^, makes it possible to measure intracellular iron oxidation and to assess the ability to maintain the intracellular reducing potential (Pelizzoni et al., 2011). In our astrocytes, whereas acute Fe^2+^ entry caused a similar fluorescence quenching in both controls and FRDA-like astrocytes (Fig. 2C), fura-2 fluorescence recovery was significantly higher in FRDA-like astrocytes (Fig. 2D), indicating a defect in the intracellular reducing potential.

### Effects of FXN downregulation on mitochondrial membrane potential

**Fig. 3.**
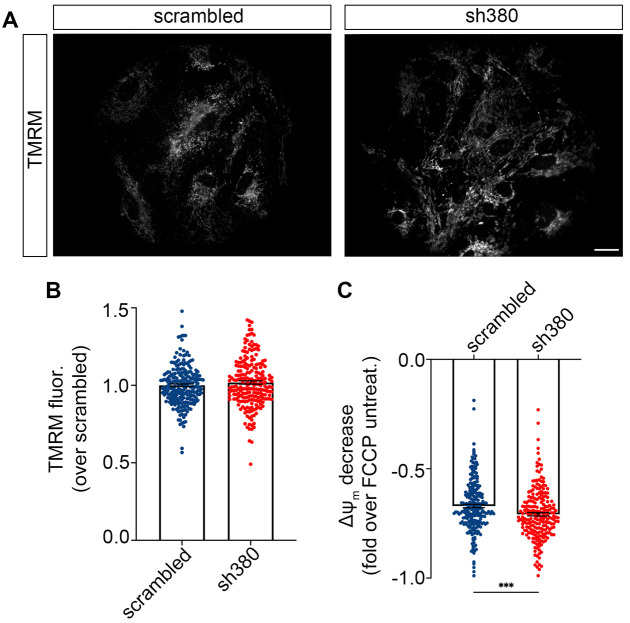
**Mitochondrial membrane potential is not affected in FRDA-like astrocytes.** (A) Representative images from three biological replicates (*n*=229 high-throughput microscopy fields for scrambled and 222 for sh380) of mitochondria loaded with TMRM, from scrambled- and sh380-transduced astrocytes. Scale bars: 50 μm. (B,C) Scrambled- and sh380-transduced astrocytes loaded with TMRM were analysed by high-throughput microscopy. After the first run of acquisition (B), astrocytes were treated with FCCP (4 μM) for 10 min and the fluorescence acquisition was repeated on the same cells (C). In C, the fluorescence decrease caused by FCCP treatment was normalized to the untreated condition. Each dot represents the fluorescence of a single cell. Data from three biological replicates are expressed as mean±s.e.m.; *n*=229 high-throughput microscopy (HTM) fields for scrambled, and 222 for sh380; ***P<0.001 (linear mixed effect model with nested random effects on optical microscopy fields within the experiment).

Given the increase in basal ROS levels in sh380-transduced astrocytes, which is often caused by a reduction of the mitochondrial membrane potential (ΔΨ_m_), we investigated this parameter using TMRM, a fluorescent lipophilic dye that accumulates in active mitochondria due to their negative membrane potential. A qualitative analysis of TMRM fluorescence revealed a comparably healthy appearance of mitochondria in FRDA-like astrocytes and in scrambled controls, without signs of mitochondria fragmentation (Fig. 3A). Surprisingly, high-throughput microscopy analysis, previously used for ROS measurements, showed that ΔΨ_m_ in FRDA-like astrocytes was slightly higher (although not significantly different) than in control cells (Fig. 3B). After the first fluorescence acquisition, the astrocytes were then treated for 10 min with FCCP, an uncoupler of the respiratory chain that dissipates the ΔΨ_m_, followed by a second round of acquisition on the same cells. The relative decrease in TMRM fluorescence induced by FCCP was significantly greater in FRDA-like astrocytes, indicating a higher proton gradient than in control astrocytes (Fig. 3C). The apparent discrepancy between the untreated and the FCCP-induced ΔΨ_m_ might be attributed to low dynamic range of TMRM fluorescence changes when close to saturation (in untreated cells). This saturation can dampen the differences between control and FRDA-like astrocytes, which are instead visible at fluorescence variations within the linear range.

### Alterations of Ca^2+^ homeostasis in FRDA cerebellar astrocytes

**Fig. 4.**
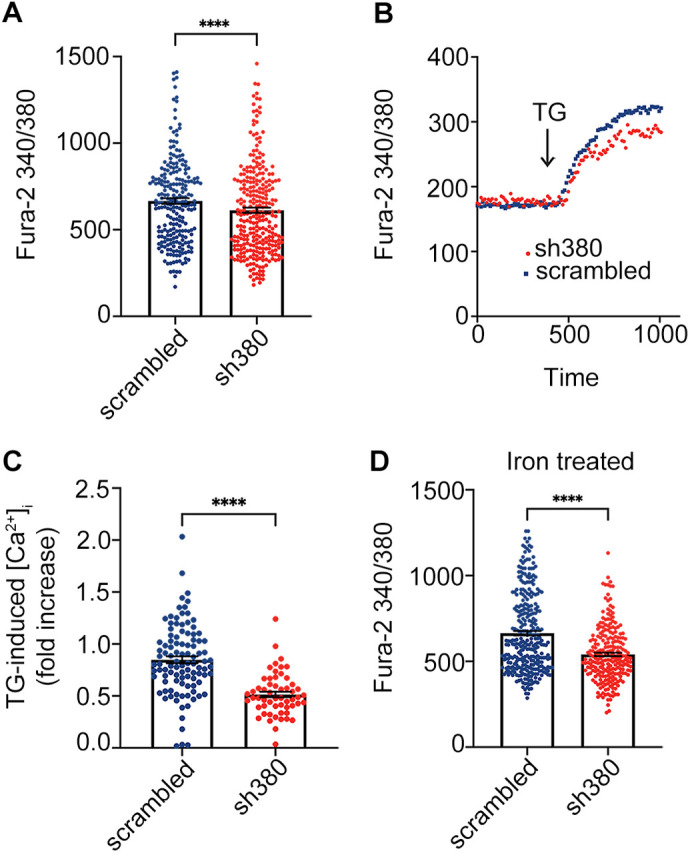
**Decreased Ca^2+^ responses in FRDA-like astrocytes.** (A) Peaks of Ca^2+^ response following acute ATP administration (100 µM) in scrambled- and sh380-transduced astrocytes. Each dot represents the maximum peak scored in each single cell. Data from five biological replicates are expressed as mean±s.e.m.; *n*=237 cells for scrambled, and 292 for sh380; ****P<0.0001. (B,C) [Ca^2+^]_i_ increase caused by Ca^2+^ store depletion following thapsigargin (TG) treatment. In B, representative kinetics of [Ca^2+^]_i_ elevation upon TG administration (1 µM). In C, TG-mediated depletion of Ca^2+^ stores. Each dot in C represents a single cell. Data, from three biological replicates are expressed as mean±s.e.m.; *n*=101 cells for scrambled and 87 for sh380; ****P<0.0001. (D) Peaks of Ca^2+^ response following ATP administration (100 µM) in cerebellar astrocytes treated for 24 h with Fe^3+^ (administered as ferric ammonium citrate, FAC, 50 μM). Each dot represents the maximum fluorescence peak scored in each individual cell. Data from five biological replicates are expressed as mean±s.e.m.; *n*=313 cells for scrambled, 237 for sh380; ****P<0.0001. Statistical analysis in A, C and D was performed with a linear mixed effect model with nested random effects on optical microscopy fields within the experiment.

Oxidative stress affects several metabolic pathways and cellular functions, with a particular impact on the complex mechanisms underlying Ca^2+^ homeostasis. To analyse Ca^2+^ metabolism in FRDA-like astrocytes, at 1 week after lentiviral infection, sh380- and scrambled-transduced astrocytes were loaded with fura-2 Ca^2+^ dye and analysed by single-cell Ca^2+^ imaging. Strikingly, neither astrocyte population responded to glutamate, whereas all cells showed a robust increase of intracellular Ca^2+^ concentration ([Ca^2+^]_i_) upon acute administration of 100 µM ATP. Surprisingly, the peak of Ca^2+^ responses induced by ATP was significantly reduced in FRDA-like astrocytes compared to that seen in scrambled controls (Fig. 4A). One of the mechanisms responsible for the amplitude of the Ca^2+^ elevation induced by metabotropic, but also ionotropic, stimuli, is the regulated release of Ca^2+^ from intracellular Ca^2+^ stores. To investigate possible alterations in this pathway, the astrocytes were exposed to thapsigargin, a blocker of SERCA pumps and, consequently, of store refilling (Tadini-Buoninsegni et al., 2018). The [Ca^2+^]_i_ increase, resulting from thapsigargin-mediated store depletion, was significantly lower in FRDA-like astrocytes compared to that in control ones, indicating an impairment of Ca^2+^ storage (Fig. 4B,C). Several reports suggest that Ca^2+^ storage and its release from intracellular stores can be affected by the oxidative environment (Ying et al., 2008), which is elevated in FRDA-like astrocytes. To investigate this aspect, we treated the cells for 24 h with Fe^3+^ (administered as ferric ammonium citrate, FAC, 50 μM), to induce a mild iron overload and increase the oxidative environment. Indeed, under this condition, the elevation of [Ca^2+^]_i_ induced by ATP was even less pronounced in FRDA-like astrocytes than in control cells (Fig. 4D).

## DISCUSSION

For many decades, research on neurodegenerative disorders has focused primarily on the intrinsic mechanisms of neuronal toxicity. However, mounting evidence demonstrates that glial cells actively contribute to pathological processes and disease progression. Astrocytes, whose physiological role in neuronal homeostasis and function is very well established, can lose their supportive properties during a neurotoxic process, and actively contribute to neurotoxicity (Gleichman and Carmichael, 2020; Stoklund Dittlau and Freude, 2024). In FRDA, the potential impact of these non-cell autonomous mechanisms on the disease has only been partially investigated.

In this study, we generated and characterized an *in vitro* model of FRDA cerebellar astrocytes to evaluate their vulnerability to FXN knockdown and their potential contribution on FRDA pathogenesis. Indeed, selective ablation of mouse *Fxn* in GFAP-expressing precursor cells causes alterations selectively in cerebellar astrocytes, but not in forebrain astrocytes, leading to severe ataxia and early death (Franco et al., 2017). This finding suggested that cerebellar astrocytes are more vulnerable to frataxin depletion than astrocytes from other brain regions. This specific cerebellar vulnerability could be partially attributed to the unique proteomic profile of astrocytes from the cerebellum, which differ from that of astrocytes in all other brain regions analysed (Prabhakar et al., 2023).

The cerebellum is characterized by astrocyte heterogeneity, resulting from a complex and still partially unclear process of glial cell proliferation and differentiation. Astroglia originate from radial glia of the ventricular neuroepithelium through direct transformation into Bergmann glia at embryonic stages and by amplification of intermediate progenitors within the cerebellar parenchyma after birth (Cerrato et al., 2018). Based on their morphological features and functional connections, cerebellar astrocytes can be classified in three main groups: the Bergmann glia, the star-shaped astrocytes (also defined as velate protoplasmic astrocytes) of the granular layer in the cerebellar cortex, and fibrous astrocytes in the white matter (Buffo and Rossi, 2013; Cerrato, 2020). Our cultures, obtained from cerebella harvested at neonatal stages, likely reflect this heterogeneity, with cells characterized by varying morphologies (stellate, fibroblast-like and elongated) and different expression of the typical astrocytic markers (GFAP and S100β). The low percentage of GFAP-positive cells found in our cultures mirrors similar behaviour observed in rat hippocampus (Walz and Lang, 1998) and cerebral cortex (Zhang et al., 2019). Within this framework, no morphological differences seem to be induced by FXN knockdown, likely because the *in vitro* differentiation programme, once initiated, is no longer affected by FXN silencing. GFAP, a typical marker that significantly increases in reactive astrocytes and during astrogliosis, was also found to be elevated in the cerebellar astrocytes where FXN was downregulated at early stages (Franco et al., 2017). However, in our cultures, under pro-inflammatory conditions, GFAP levels sharply decreased compared to those in resting conditions (C.M., L.C., F. Codazzi, unpublished data). Again, this behaviour does not depend on FXN depletion, but it should deserve attention when evaluating the effects of inflammatory conditions occurring during FRDA progression (Hayashi et al., 2014; Koeppen et al., 2016).

Having obtained an FRDA-like cerebellar astrocytic model, showing a decrease in FXN levels comparable to that observed in individuals with FRDA, we investigated the functional alterations caused by FXN downregulation. Impaired mitochondrial metabolism and a defective respiratory chain, associated with ROS production, are considered one of the main mechanisms of FRDA pathogenesis (Calabrese et al., 2005; Lupoli et al., 2018). Indeed, a typical hallmark of FRDA is a reduction in ΔΨ_m_, observed in affected neurons as well as in various cell types. However, our FRDA-like astrocytes do not display noticeable morphological changes in mitochondria, nor a significant alteration in ΔΨ_m_. Interestingly, this parameter appears slightly higher, although not significantly different, in sh380-transduced astrocytes compared to that for scrambled-transduced astrocytes. Accordingly, the effect of FCCP, an uncoupler of the respiratory chain that disrupts the proton gradient, was more pronounced in FRDA-like astrocytes than in the control counterpart. Our results are consistent with data obtained from cerebellar cultures, where the ΔΨ_m_ was significantly decreased in granule cells of FRDA YG8R mice, but not in glial cells, where this parameter was comparable to cultures from control mice (Abeti et al., 2016). This finding suggests a compensatory mechanism in glial cells that maintains normal ΔΨ_m_ under FXN depletion.

With respect to the observed increase in oxidative stress, the higher production of free radicals, typically observed in FXN-deficient cells and associated with oxidative damage in FRDA (Codazzi et al., 2016), depends on the reduction of ΔΨ_m_. Nonetheless, our FRDA-like astrocytes showed higher levels of basal ROS production, despite the unchanged ΔΨ_m_. However, it has been shown that FXN intrinsically increases cellular antioxidant defences by activating glutathione peroxidase and elevating reduced thiols (Shoichet et al., 2002). Therefore, the downregulation of FXN accounts for higher oxidative stress in FRDA-like astrocytes. It is important to note that ROS generation in FRDA-like astrocytes is not excessively elevated, likely due to their high ability to buffer perturbation of various intracellular and extracellular parameters. Indeed, our cerebellar FRDA-like astrocytes consume their GSH content to counteract the basal ROS elevation. However, GSH depletion impairs their ability to maintain the redox cellular balance, as observed under a condition of acute iron overload (Pelizzoni et al., 2011), making the astrocytes more vulnerable to oxidative insults. This condition also impairs the protective role of astrocytes towards neuronal cells, which depend entirely on astrocytes for their GSH content (Aschner, 2000).

Cerebellar FRDA-like astrocytes also display alterations in Ca^2+^ homeostasis that might affect the physiological functions of astrocytes. Astrocytes generate complex Ca^2+^ signals, such as Ca^2+^ oscillations and waves (Codazzi et al., 2001), in response to neurotransmitter spillover from the synaptic cleft and to other extracellular signals. In turn, the spatiotemporal aspects of Ca^2+^ dynamics permit a controlled release of active molecules and neuromodulators, both locally and within long-range neuro-astrocytic networks. Spontaneous and repetitive Ca^2+^ waves have been observed and characterized in Bergmann glia *in vivo* (e.g. Asemi-Rad et al., 2024; Hoogland and Kuhn, 2010). These Ca^2+^ signals seem only partially mediated by glutamatergic AMPA receptors, whose Ca^2+^ permeability depends on the expression of the Glu-R2 subunit. Likewise, the stimulation of purinergic receptors, particularly the metabotropic P2Y family, promotes complex Ca^2+^ responses (Hoogland and Kuhn, 2010). Our data confirm a lack of Ca^2+^ signals induced by glutamate stimulation (data not shown) whereas the responses to ATP are consistent. Moreover, we demonstrate that ATP-induced Ca^2+^ responses in FRDA-like astrocytes are lower in comparison to controls, and that a lower Ca^2+^ loading of intracellular stores (revealed by thapsigargin-induced store depletion) accounts for Ca^2+^ mishandling in FRDA-like astrocytes. However, we cannot rule out the possibility that an increased mitochondrial re-uptake of Ca^2+^ might contribute to the decreased peak in ATP responses. A similar impairment of the ability to load Ca^2+^ in intracellular stores has been observed in cerebellar granule neurons derived from the YG8R FRDA mouse model (Abeti et al., 2018). This altered Ca^2+^ homeostasis can be caused by the higher level of oxidative stress displayed by FRDA-like astrocytes, due to the presence of cysteine residues in SERCAs that are sensitive to oxidative environments (Ying et al., 2008). Moreover, although it is more common to observe elevated Ca^2+^ responses under toxic cellular conditions, a similar decrease of Ca^2+^ signals has been observed in other neurodegenerative models. For instance, in cellular models of TDP-43 (also known as TARDBP) proteinopathy, a feature of amyotrophic lateral sclerosis (ALS), glutamate-elicited Ca^2+^ responses were significantly lower than in control cells (Pisciottani et al., 2023). Likewise, retinal Müller cells maintained in high glucose medium, to mimic diabetic retinopathy, also show reduced Ca^2+^ responses to ATP stimulation compared to that for cells maintained in normal glucose medium (Rosato et al., 2022). Similarly, in astrocytes, the activation of P2Y receptors enhances resistance to oxidative stress by a Ca^2+^-dependent increase of mitochondrial metabolism (Wu et al., 2007). Therefore, lower Ca^2+^ responses can impair this protective pathway. Additionally, the release of several active molecules and factors by astrocytes depends on Ca^2+^ elevation, so impaired responses to ATP could affect and alter their physiological interplay with neuronal cells.

### Conclusions

In this study, we characterized for the first time a set of functional alterations in FRDA-like cerebellar astrocytes. Although FXN downregulation does not seem to cause macroscopic morphological changes, in-depth analyses revealed modification of some cellular parameters that might significantly affect long-term neuronal function and synaptic activity. The reduction of astrocytic GSH might indeed compromise the already low antioxidant capacities of neurons. This might accelerate disease progression, increasing the neurotoxic impact of iron accumulation and oxidative stress. Moreover, the reduced Ca^2+^ responses to purinergic stimuli might alter the ability of astrocytes to release molecules that actively modulate and regulate synaptic function over long distances, further contributing to neuronal dysfunction.

The most overtly affected neurons in the brain are the large glutamatergic neurons of the dentate nuclei, which are not easily co-cultured with FRDA-like astrocytes. Different *in vitro* models (e.g. organotypic cerebellar slices maintained with medium conditioned by astrocytes) will make it possible to assess how FXN depletion in cerebellar astrocytes might alter their complex interplay with glutamatergic neurons and contribute to neuronal dysfunction. Likewise, selective stereotaxic injection of adeno-associated viral particles into the mouse lateral nucleus (e.g. Asemi-Rad et al., 2024) to achieve cerebellar astrocyte-specific FXN knockdown would provide a useful *in vivo* model of locally altered astrocyte–neuron interactions and of their impact on the ataxic phenotype.

## MATERIALS AND METHODS

### Primary culture of cerebellar astrocytes

Animal handling and experimental procedures were performed in accordance with the EC guidelines (EC Council Directive 86/6091987) and with the Italian legislation on animal experimentation (Decreto L.vo 116/92) and approved by our Institutional Animal Care and Use Committee.

Mouse cerebella (strain C57BL/6N) were harvested at postnatal day 1 (P1) or 2 (P2) and digested with 2.5 mg/ml trypsin (Sigma-Aldrich, cat. T1005-1G) and 1.5 mg/ml DNase (EMD Millipore, cat. 260913-10MU). Subsequently, cerebella were mechanically dissociated to obtain a single cell suspension and centrifuged at 100 ***g***. Pellets were resuspended in astrocyte culture medium [1× MEM alpha medium plus GlutaMAX^TM^ (Gibco, cat. 32561-029), 10% fetal bovine serum (EuroClone, cat. ECS5000L), 1% penicillin-streptomycin (EuroClone, cat. ECB3001D) and 33 mM glucose (D-glucose; Sigma-Aldrich, cat. G5767-500G)], plated on plastic treated with poly-L-lysine (100 µg/ml, Sigma, cat. P1274) and cultured in an incubator at 37°C and 5% CO_2_. After 2–3 passages, cerebellar astrocytes were plated either on plastic or glass coverslips, both treated with poly-L-lysine, depending on experimental requirements.

### Production of lentiviruses expressing *Fxn*-specific and scrambled sh-RNAs

To downregulate the *Fxn* gene in cerebellar astrocytes, vectors encoding for either TRCN0000178380 (5′-GACTTGTCTTCATTGGCCTAT-3′), named sh380, or TRCN0000198535 (5′-GAGTTCTTTGAAGACCTCGCA-3′), named sh535, were generated; plkO.1 SHC002 (5′-ATCTCGCTTGGGCGATAGTGC-3′) named scrambled, was used as control. These vectors were engineered in the laboratory starting from pLKO.1-TRC (Sigma-Aldrich) cloning vectors carrying the shRNA sequence (Sigma-Aldrich) expressed under the control of the hU6 promoter; the puromycin resistance sequence was replaced with the sequence encoding for GFP.

Lentiviral particles were prepared as described previously (Amendola et al., 2005). Using the Ca^2+^-phosphate precipitation method, HEK293T cells (ATTC, CRL-3216) were transiently co-transfected with the transfer vectors, the MDLg/pRRE plasmid, the RSV-Rev plasmid and the MDLg plasmid (kind gifts from Luigi Naldini, San Raffaele Telethon Institute for Gene Therapy, Milan, Italy). After 72 h of transfection, cell supernatants containing lentiviral particles were collected, filtered and ultracentrifuged (20,000 rpm, 2 h, SW32Ti rotor). The pellets were resuspended, divided into aliquots and stored at −80°C. To calculate the transduction efficiency, GFP-positive cells and the total number of cells were counted.

Cerebellar astrocytes were plated on the appropriate support and infected once they reached the desired confluence. The experiments were performed 7 days after the infection.

### Western blotting

Cerebellar astrocytes were scraped on ice with lysis buffer (0.1 M EDTA, 2% NP-40, 0.2% SDS and CLAP (final concentration: 16 μM chymostatin, 21 μM leupeptin, 16 μM antipain, 15 μM pepstatin; Sigma-Aldrich). Protein extracts were quantified with Pierce™ BCA Protein Assay kit (Thermo Fisher Scientific, 23225). Western blotting was performed as described in Guarino et al. (2022). Briefly, 30 μg of protein lysate were suspended in Laemmli sample buffer (final concentration: 50 mM Tris-HCl pH 6.8, 2.5 mM EDTA/Na, 2% SDS, 5% glycerol, 0.2 M dithiothreitol and 0.01% Bromophenol Blue), denatured for 5 min at 95°C, loaded onto a 12.5% polyacrylamide gel and then transferred onto nitrocellulose membrane. After 1 h of incubation at room temperature (RT) with blocking buffer [10 mM Tris-HCl, 150 mM NaCl, 0.1% Tween-20 (TBST) containing 5% skimmed powdered milk], membranes were incubated overnight at 4°C with primary antibodies (see below) diluted in blocking buffer and, after extensive washing, with horseradish peroxidase-conjugated anti-rabbit-IgG, anti-mouse-IgG or anti-chicken-IgY secondary antibody (Bio-Rad, Hercules, CA, USA). Proteins were revealed by direct acquisition using the Bio-Rad ChemiDoc™ MP Imaging system by Super Signal West Chemiluminescent Substrate (Thermo Fisher Scientific). For loading controls, membranes were stripped in a stripping buffer (0.2 M glycine, 0.1% SDS, 1% Tween-20, pH 2.2) and re-probed with the appropriate antibody. Quantification was performed with Image Lab™ Software (Bio-Rad) and protein levels normalized against the loading control (α-tubulin). The ChemiDoc™ outputs relative to all six biological replicates are shown in Fig. S2.

Primary antibodies were against: α-tubulin (1:6000, Merck, cat. T9026), GFP (1:3000, Abcam, cat. ab13970) and FXN (1:1000, Merck, cat. AB15080); diluted in 5% Milk in TBST.

### RT-qPCR

RNA was extracted from cells using TRIzol™ Plus RNA Purification kit (Invitrogen, cat. 12183555) and quantified using Nanodrop (ThermoFisher Scientific). 1 µg of RNA was retro-transcribed with M-MLV Reverse Transcriptase (Invitrogen, cat. 28025013). RT-qPCR was carried out with LightCycler480 (Roche) using LightCycler480 SYBR Green I Master Mix (Roche).

The primers used for transcript quantifications are: FXN forward primer 5′-TCACCATTAAGCTGGGCG, reverse primer 5′-TTCTTCCCGGTCCAGTCATA; β-actin forward primer 5′-CTGTCGAGTCGCGTCCACC, reverse primer 5′-TCGTCATCCATGGCGAACTG.

The experiment was performed on biological triplicates, and RNA levels were normalized to the level of transcript coding for β-actin.

### Fluorescence microscopy setup

A video imaging setup consisting of an Axioskop 2 microscope (Zeiss, Oberkochen, Germany) and a Polychrome IV light source (Till Photonics, GmbH, Martinsried, Germany) was used for single-cell experiments. Fluorescence images were collected by a cooled CCD videocamera (PCO Computer Optics GmbH, Kelheim, Germany). The ‘Vision’ software (Till Photonics) was used to control the acquisition protocol and to perform data analysis (Rosato et al., 2022; Vangelista et al., 2005). This instrument was used for fura-2-based Ca^2+^ analyses, mBCl-based GSH measurements and acute iron overload experiments.

The automated ArrayScan XTI platform (Thermo Fisher Scientific) was used for reactive oxygen species (ROS) and TMRM-mitochondrial membrane potential analyses. This is referred to as high-throughput microscopy (HTM) in the results section.

### Dye loading and treatments

Dye loading and single-cell experiments were performed in Krebs Ringer HEPES buffer (KRH, containing 5 mM KCl, 125 mM NaCl, 2 mM CaCl_2_, 1.2 mM MgSO4, 1.2 mM KH2PO4, 6 mM glucose and 20 mM Hepes, pH 7.4). Experiments were performed at room temperature. Fluorescent dyes (from Molecular Probes, Thermo Fisher Scientific, when not specified) were loaded as described in the dedicated paragraphs. After dye loading, cells were washed twice with fresh KRH and analysed in the same buffer.

An acute iron overload protocol was performed by incubating cells for 2 min with 20 μM pyrithione (an iron ionophore that allows a kinetically controlled Fe^2+^ entry), before the administration of 100 μM Fe^2+^ (as FAS, ferrous ammonium sulfate) for 3 min, followed by several washes with KRH. To monitor Fe^2+^ entry and iron oxidative status, fluorescence quenching and de-quenching of fura-2 was evaluated at excitation wavelength of 356 nm, the Ca^2+^-insensitive wavelength in our optical system. Chronic iron overload was performed by incubating the cells overnight with 100 μM Fe^3+^ (as ferric ammonium citrate, FAC).

### Analysis of ROS

Astrocytes were plated on a 96-well plate, transduced with lentiviral particles and analysed using the ArrayScan XTI platform (Thermo Fisher Scientific) 1 week later. Cells were loaded with CellROX Orange Reagent (5 μM, 30 min at 37°C), to analyse ROS accumulation in the mitochondria, and with Hoechst 33342 (5 min at room temperature, 2 μg/ml final concentration) for nuclear staining.

### Analysis of reduced glutathione levels

Reduced glutathione (GSH) content was measured at single-cell level with the thiol-reactive fluorescent probe monochlorobimane (mBCl; Sigma-Aldrich); mBCl turns fluorescent after conjugation with GSH. In the mBCl assay, the astrocytes are visualized by means of a nucleic acid stain with SYTO™ (1 μM, incubated 10 min at 37°C); thereafter 50 μM of mBCl was added to KRH buffer at the beginning of the experiments and the kinetics of fluorescent GSH-monochlorobimane adduct formation was analysed for 20 min, until the plateau phase was reached.

### Mitochondrial membrane potential measurements

Astrocytes were plated on a 96-well plate, transduced with lentiviral particles and analysed using the ArrayScan XTI platform 1 week later. Cells were loaded with tetramethylrhodamine methyl ester (TMRM; 15 min at 37°C, 25 nM final concentration) in the presence of 2 μM Cyclosporin H (Cayman Chemical); the solution was maintained in the bath during image acquisition. Subsequently, 4 μM trifluoro carbonyl cyanide phenylhydrazone (FCCP) was added in each well and fluorescence intensity was reacquired on the same cells.

### Ca^2+^ measurement

Cerebellar astrocytes were loaded with fura-2 acetoxymethyl ester (AM, Calbiochem; 40 min at 37°C, 4 μM final concentration). Images were collected by a Axioskope-2-based setup with a rate of 1 ratio image every 2 s. The ‘Vision’ software (Till Photonics) was used to control the acquisition protocol and to perform data analysis.

### Immunofluorescence

Astrocytes were plated on a 96-well plate, transduced with lentiviral particles and analysed using the ArrayScan XTI platform after immunofluorescence staining. Cerebellar astrocytes were fixed with 4% paraformaldehyde (PFA) in PBS for 15 min. The fixed cells were incubated with primary antibodies (see below) in 10% goat serum (GS), 0.1% Triton X-100 in PBS overnight at 4°C and, subsequently, with anti-rabbit-IgG or anti-mouse-IgG secondary antibodies (1:1000, Invitrogen), and counterstained with DAPI (D9542, Sigma).

Images were acquired using a Axio Observer (Zeiss Axio Observer.Z1 with Hamamatsu EM9100) and ArrayScan microscope. Quantification of cell types was performed with ImageJ-Fiji or ArrayScan software.

Primary antibodies used were against: glial fibrillary acidic protein (GFAP 1:100, DakoCytomation, cat. Z0334); S100 (β-subunit) (1:500, Sigma-Aldrich, cat. S2532).

### Statistical analysis

Data are expressed as mean±s.e.m. of at least three independent experiments. For data in Figs 1 and 2A and Fig. S1, statistical analysis was conducted using GraphPad Prism, as reported in the figure legends. For data in Figs 2B,D, 3, 4 and Fig. S2, statistical analysis was conducted using the R software (R Project for Statistical Computing; https://www.r-project.org/) applying the linear mixed effect model with nested random effects on optical microscopy fields within the experiment. Differences yielding a *P*≥0.05 were regarded as non-significant.

## Supplementary Material

10.1242/joces.263446_sup1 Supplementary information
