# Supplementary material for: Altered Ca2+ responses and antioxidant properties in Friedreich's ataxia-like cerebellar astrocytes
Source: J Cell Sci. 2025 Jan 9;138(1):jcs263446. doi: 10.1242/jcs.263446 (PMC11828468; doi:10.1242/jcs.263446)
Supplement: Supplementary information [file joces-138-263446-s1.pdf]

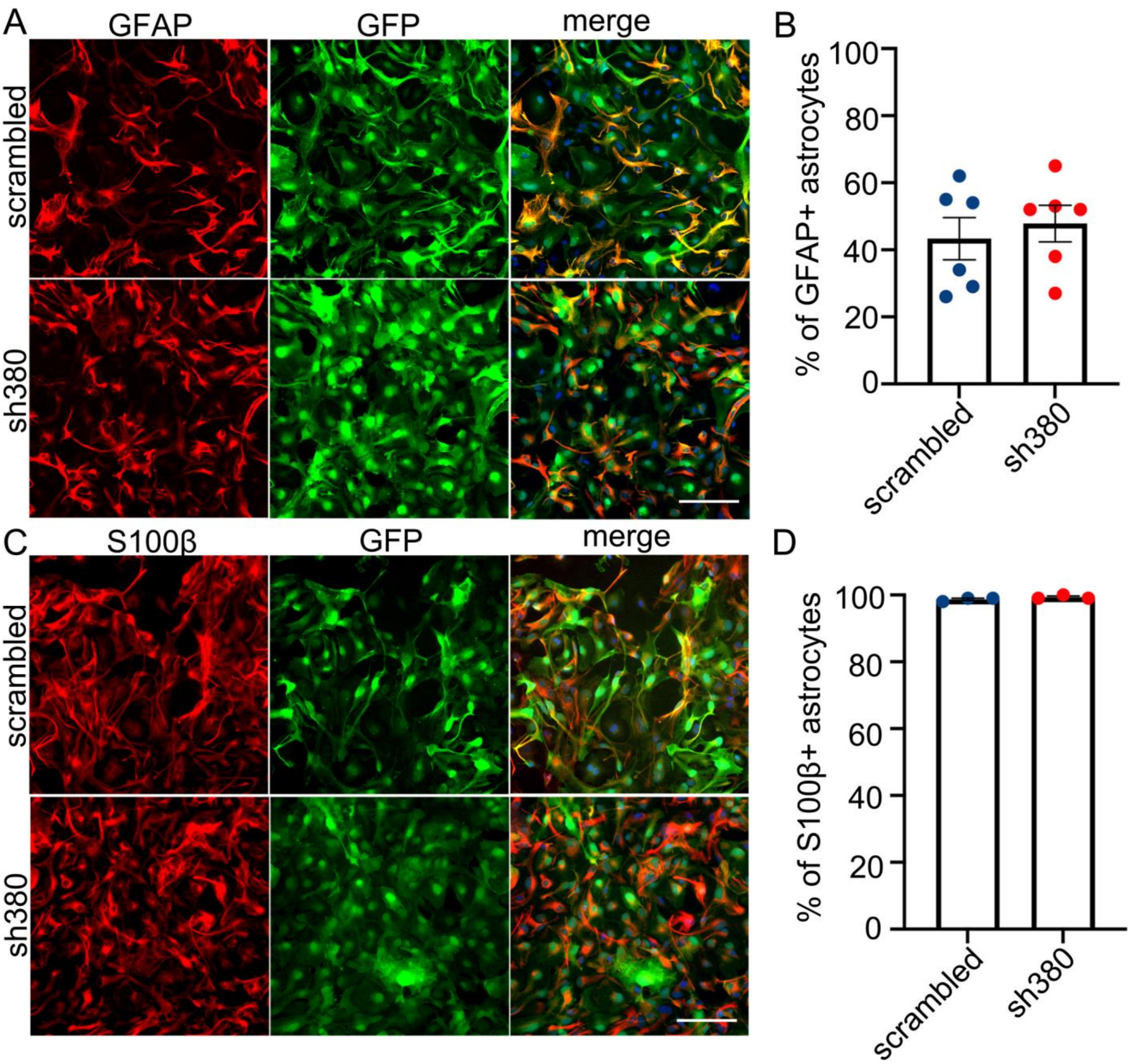

Fig. S1. No differences in astroglial marker expression between FRDA-like and control astrocytes

A: Scrambled- and sh380-transduced astrocytes immunostained with an antibody detecting glial fibrillary acidic protein (GFAP, red), 7 days after transduction. Size bar: 50 µm

B: Percentage of GFAP+ astrocytes, over the total number of cells in the field (DAPI+ cells), analyzed by high-throughput microscopy. Each dot represents the average of a single culture well. Data, from 3 biological replicates, are expressed as mean +/- SEM; n=5801 cells for scrambled, and 5669 for sh380

Journal of Cell Science • Supplementary information

C: Scrambled- and sh380-transduced astrocytes immunostained with an antibody detecting 8100 (red), 7 days after transduction. Size bar: 50 µm

D: Percentage of S100+ astrocytes, over the total number of cells in the field (DAPI+ cells), analyzed by high-throughput microscopy. Each dot represents the average of a single culture well. Data from 3 biological replicates are expressed as mean +/- SEM; n=4316 cells for scrambled, and 3352 for sh380.

Journal of Cell Science • Supplementary information

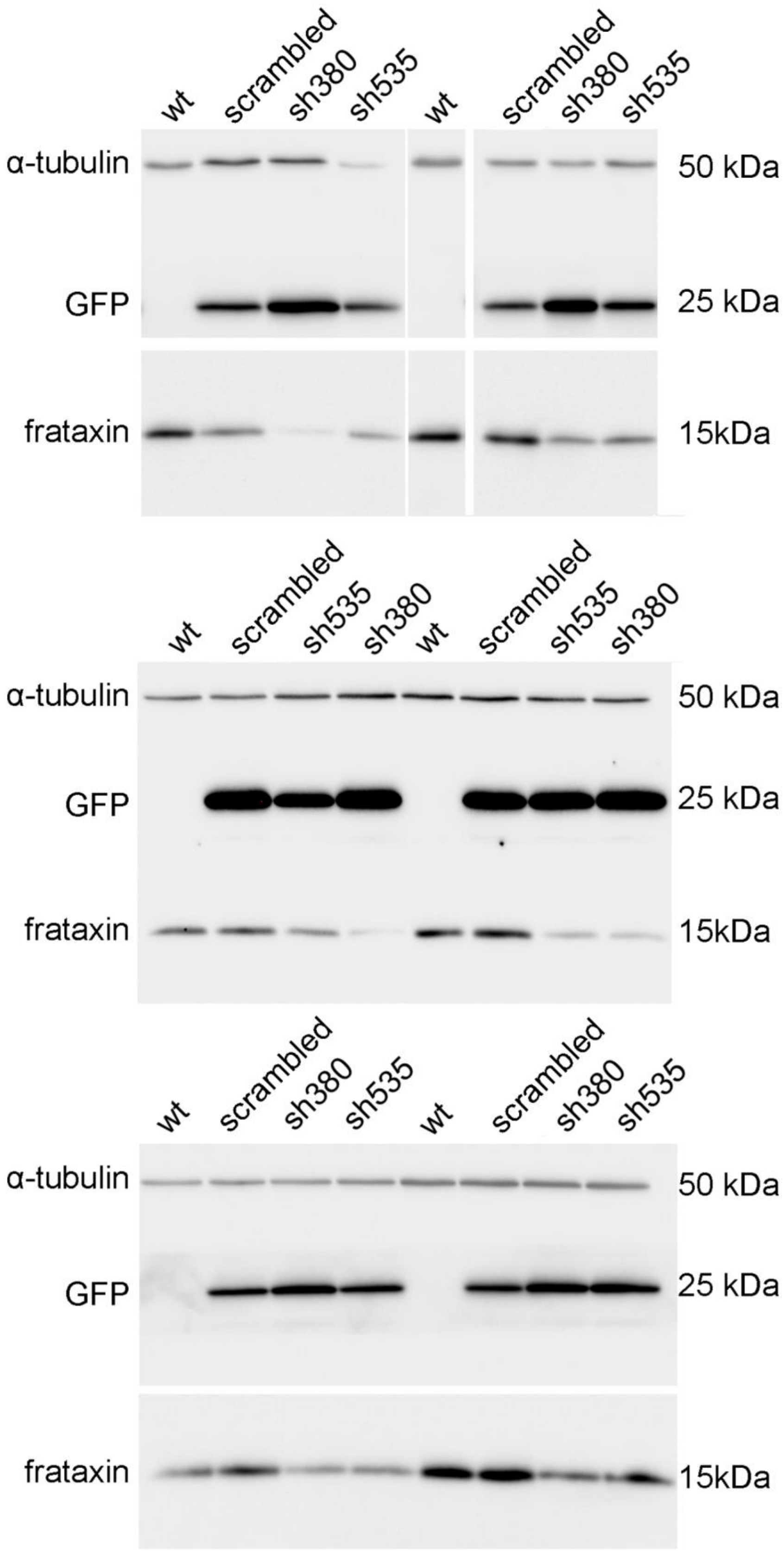

Fig. S2. Blot transparency

Chemidoc™ outputs relative to six biological replicates of western blot of protein lysates derived from wt, scrambled-, sh535- and sh380- transduced astrocytes, immunostained with antibody detecting FXN (15kDa). GFP (25kDa) was used to assess the transduction efficiency; a-tubulin (50kDa) was used as a loading control.

Journal of Cell Science • Supplementary information
